# Vegetation Changing Patterns and Its Sensitivity to Climate Variability across Seven Major Watersheds in China

**DOI:** 10.3390/ijerph192113916

**Published:** 2022-10-26

**Authors:** Qin Wang, Qin Ju, Yueyang Wang, Quanxi Shao, Rongrong Zhang, Yanli Liu, Zhenchun Hao

**Affiliations:** 1 State Key Laboratory of Hydrology-Water Resources and Hydraulic Engineering, Hohai University, Nanjing 210098, China; 2 CSIRO Data 61, Australian Resources Research Centre, Kensington, WA 6151, Australia; 3 State Key Laboratory of Hydrology-Water Resources and Hydraulic Engineering, Nanjing Hydraulic Research Institute, Nanjing 210029, China

**Keywords:** vegetation variations, climate change, regionalization, Vegetation Sensitivity Index (VSI)

## Abstract

Climate changes have profound impacts on vegetation and further alter hydrological processes through transpiration, interception, and evaporation. This study investigated vegetation’s changing patterns and its sensitivity to climate variability across seven major watersheds in China based on a hybrid regionalization approach and a novel, empirical index—Vegetation Sensitivity Index (VSI). Vegetation showed linearly increasing trends in most of the seven watersheds, while decreases in vegetation were mostly found in the source regions of the Yangtze River Basin (YZRB) and Yellow River Basin (YRB), the forest and grassland areas of the Songhua River Basin (SHRB) and Liao River Basin (LRB), the Yangtze River Delta, and the Pearl River Delta during the growing season. The selected watersheds can be categorized into 11 sub-regions, and the regionalization result was consistent with the topography and vegetation types; the characteristics of vegetation dynamics were more homogeneous among sub-regions. Vegetation types such as forests and shrubland in the central parts of the YZRB were relatively more vulnerable to climate variations than the grasslands and alpine meadows and tundra (AMT) in the source regions of the YZRB and YRB and the Loess Plateau of the YRB. In arid and semi-arid regions, precipitation had a profound impact on vegetation, while, at low latitudes, solar radiation was the main controlling factor. Such comprehensive investigations of the vegetation–climate relationship patterns across various watersheds are expected to provide a foundation for the exploration of future climate change impacts on ecosystems at the watershed scale.

## 1. Introduction

Vegetation is an important regulator of terrestrial carbon cycles, energy exchange, and water balance [1,2,3]. It alters hydrological processes through transpiration, interception, and evaporation, resulting in fluctuations in the rainfall–runoff relationship and runoff dynamics [4,5,6,7,8]. Duethmann and Blöschl [6] analyzed changes in evaporation estimated for 156 catchments in Austria and suggested that 34 ± 14% of the observed increase in catchment evaporation may be attributed to increased vegetation activity. Koch et al. [8] found that vegetation shifts in the Arctic will deliver water back to the atmosphere and to subsurface aquifers, and then substantially reduce discharge in headwater streams. Hrachowitz et al. [9] demonstrated that deforestation will reduce the vegetation-accessible water storage capacity, which affects catchment travel time distributions. Thus, understanding vegetation dynamics at the watershed scale can benefit the planning, management, and sustainable development of water resources.

The functioning of the Earth’s ecosystems is significantly impacted as a consequence of atmospheric CO_2_ concentrations and other climatic drivers, changing under the unprecedented climatic changes occurring in the 21st century [10,11]. With large-scale, high-precision remote sensing datasets, the considerable response of vegetation to climate changes at regional and global scales has been thoroughly established. Firstly, plant phenology and biome distributions have been altered by climate change [12]. Secondly, an increase in greening and the productivity of vegetation was detected under the warming climate [13,14]. In contrast, concomitant regional extreme heatwaves and drought events can stifle vegetation growth [15]. Additionally, climate changes, combined with increasing atmospheric CO_2_ enrichment and nitrogen (N) fertilization, can substantially enhance the photosynthetic efficiency of vegetation and accelerate peak growth [14,16]. In general, precipitation, temperature, and solar radiation have been considered as the three major climatic drivers, which account for more than half of the terrestrial vegetation variability [17]. In the middle–high latitudes of the Northern Hemisphere, temperature alters the photosynthetic activity onset, termination, and performance [18,19]. Increasing precipitation may benefit vegetation growth in arid and semi-arid areas with water deficits [20,21], while excessive precipitation would limit vegetation growth by causing a reduction in radiation and temperature in humid areas [17]. In tropical rainforests, vegetation is more sensitive to radiation [17]. On the other hand, due to the biophysical reactions in plant respiration, photosynthesis, and evapotranspiration, vegetation fluctuations also relate to climate change [22,23,24,25,26]. Forzieri et al. [25] demonstrated that the control of vegetation on global terrestrial energy fluxes increased during 1982–2016. Kafy et al. [24] used machine learning algorithms to predict urban thermal conditions in Cumilla, and found that the reduction in vegetation cover significantly increased the urban heat island effect. Rahaman et al. [22] also claimed that forest cover loss in Penang city caused an increase in the average temperature of 13 °C over 25 years. In general, the underlying mechanisms between climate changes and vegetation dynamics are non-linear and complex [27,28].

Nowadays, vegetation dynamics and strong associations between vegetation and climate change have been reported in various watersheds of the world [29,30,31,32,33,34,35]. Olivares et al. [34] reviewed the impact of climate change on lowland rainforests in the Amazon basin, and concluded that warming will limit plant species survival by decreasing vegetation productivity. Alhumaima and Abdullaev [33] found that March/April vegetation was strongly correlated with October–March precipitation and January–March temperatures in the lower Tigris basin. Morgan et al. [30] discovered that climate variability is a short-term driver of vegetation changes, and human influence had a long-term effect, in the Lake Victoria Basin. Furthermore, vegetation changes and its response to climate changes at the watershed scale have also received a great deal of attention in China [3,36,37]. For example, in the Yellow River Basin and Yangtze River Basin, vegetation variations were found to be spatially heterogeneous, and climate changes had a great influence on vegetation, especially in the central area [38]. The temperature made a determining contribution to vegetation greening in the Yangtze River Basin, while solar radiation was a strong, negative determining factor, and the correlation between precipitation and vegetation was low due to the abundant water [38,39,40]. For river basins in the Tibetan Plateau, such as the Yarlung Zangbo River Basin and the Three-River Headwaters region, precipitation was identified as the critical climatic factor in vegetation variations, but the relationship between vegetation and temperature varied with aridity [3,41,42]. The effect of precipitation on vegetation in arid mountain–oasis river basins in Northwest China was strong and varied spatially with the precipitation pattern [36], while the relationships between vegetation and climatic variations differed by vegetation type in the Amur-Heilongjiang River Basin [43]. According to previous studies, vegetation changes and their responses to climatic change can be expected to have spatial heterogeneity due to the complex spatial patterns in climatic zones, ecosystem types, biotopes, and plant species [44].

However, these studies still have several gaps. First, the results from previous studies on vegetation changes are mostly based on pixel scales or regional averaging, without considering spatial patterns. In this case, the spatial heterogeneity in vegetation trends and corresponding driving factors at watershed scales need a deeper understanding. Second, previous assessments on vegetation ecosystems that respond to climate change mostly focused on investigating the mean climate state, ignoring the variability in climate [45], which will limit the characterization of their relationship. To tackle the research gaps mentioned above, this paper used a hybrid regionalization approach to analyze the spatial heterogeneity of vegetation changes across watersheds in China and reveal the distribution of vegetation sensitivity to climate variability by a novel, empirical method, the Vegetation Sensitivity Index (VSI). Three steps were taken: (1) explore the vegetation dynamics of seven major watersheds in China during the growing season from 1982 to 2015; (2) distinguish homogenous regions of vegetation change in seven major watersheds across China through a hybrid regionalization approach; (3) quantify the sensitivity of vegetation to climate variability using the VSI on pixel and regional scales. Overall, this study provides new perspectives on vegetation changes and the triggering mechanism of climate change, which is valuable for the prediction of future vegetation dynamics and for the sustainable development of water resources.

## 2. Materials and Methods

### 2.1. Study Area

**Figure 1:**
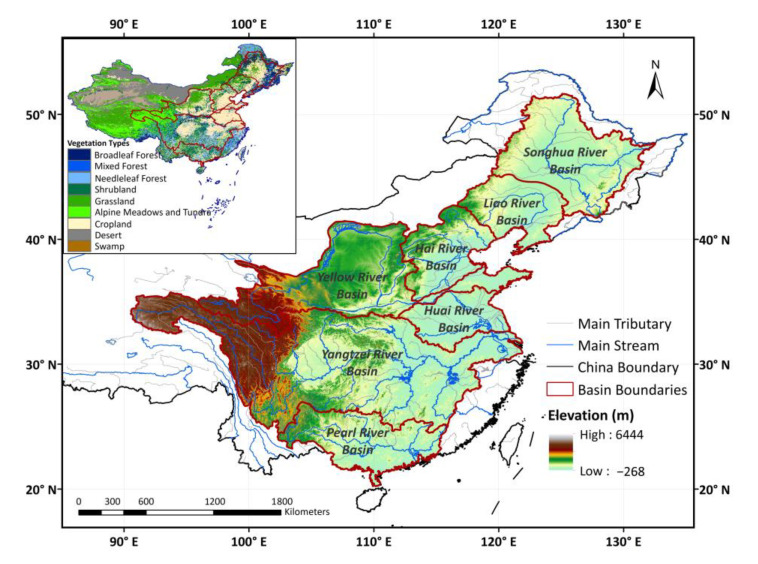
Spatial distribution of vegetation types and locations of seven major watersheds in China.

Spanning different climatic conditions, seven major watersheds were chosen to investigate the changing patterns of vegetation and its sensitivity to climate variability across watersheds in China, including the Songhua River Basin (SHRB), Liao River Basin (LRB), Hai River Basin (HaiRB), Yellow River Basin (YRB), Yangtze River Basin (YZRB), Huai River Basin (HuaiRB), and Pearl River Basin (PRB). The total basin area of the seven major watersheds is 451.19 × 10^4^ km^2^, covering 47% of China’s total land area, and the total population of the seven major watersheds constitutes more than 80% of China’s total population [46]. A map of the vegetation types and locations, and detailed information about the selected watersheds, is given in Figure 1 and Table 1, respectively.

### 2.2. Datasets and Processing

The normalized difference vegetation index (NDVI), acquired from a biweekly NDVI dataset developed by the Global Inventory Monitoring and Modeling Studies (GIMMS) group with a spatial resolution of 1/12° from 1981 to 2015 (http://ecocast.arc.nasa.gov/data/pub/gimms (accessed on 12 February 2020)), has been commonly used to describe vegetation changes at regional or global scales [40,47]. To lessen the impact of clouds and aerosols, the maximum value composite technique was used to produce monthly NDVI observations from biweekly data [43,48]. Moreover, our study was limited to the growing season (April to October for the whole study area) to eliminate the impact of winter snow [42,47]. However, it should be noted that the actual growing season may differ among watersheds. After this, pixels with an annual growing season NDVI (GS-NDVI) less than 0.1 were designated as bare ground and removed.

Monthly gridded climate data (mean monthly air temperature, precipitation, and solar radiation), with a spatial resolution of 0.1° × 0.1°, were derived from the China Meteorological Forcing Dataset (CMFD, https://data.tpdc.ac.cn/en/data/8028b944-daaa-4511-8769-965612652c49 (accessed on 20 February 2020)) [49,50]. This gridded reanalysis climate dataset has a high level of precision and has been employed to conduct climate-change-related research across China [45,51]. The growing season’s climate data were used and further resampled to 1/12° to match the resolution of NDVI through the bilinear interpolation method. In addition, the spatial distribution of vegetation types was acquired from the Resource and Environment Science and Data Center in China (RESDC, https://www.resdc.cn (accessed on 8 March 2020)) at a scale of 1:1,000,000, with nine vegetation types (broadleaf forest (BF), broadleaf and needleleaf mixed forest (MF), needleleaf forest (NF), shrubland, grassland, alpine meadows and tundra (AMT), cropland, swamp, and desert).

### 2.3. Methods

#### 2.3.1. Trend Analysis

The linear least-square regression [52] method was used to detect trends of vegetation and climatic drivers at both pixel scale and regional scale during the growing season from 1982 to 2015. The Theil–Sen median analysis combined with the Mann–Kendall test was used to evaluate the statistical significance [28]. According to previous studies [38,53] and the real condition of the NDVI in the YZRB, statistically significant results were categorized into five groups: significantly improved (S_NDVI_ ≥ 0.0005, Z ≥ 1.96), slightly improved (S_NDVI_ ≥ 0.0005, −1.96 ≤ Z ≤ 1.96), stable (−0.0005 ≤ S_NDVI_ ≤ 0.0005), slightly degraded (S_NDVI_ ≤ −0.0005, −1.96 ≤ Z ≤ 1.96), and significantly degraded (S_NDVI_ ≤ −0.0005, Z ≤ −1.96).

#### 2.3.2. Hybrid Regionalization Approach

A hybrid regionalization approach [54,55] was used to identify homogenous regions of vegetation change in seven major watersheds. During the regionalization procedure, the number of clusters and the initial clustering centers with the maximum values of rotated loading vector (RLVs) were initially determined by Varimax Rotated Empirical Orthogonal Functions (REOF). Then, depending on the cluster groups and centroids produced from REOF, K-means clustering analysis was used to identify homogeneous regions. After this, the final sub-regions were obtained by merging and splitting the clusters according to the geographical locations of pixels. The detailed steps of spatial regionalization are listed in Wu et al. [55].

#### 2.3.3. Vegetation Sensitivity Index

The Vegetation Sensitivity Index (VSI), developed by Seddon et al. [56], was used in this study to assess vegetation sensitivity to climate variability in seven major watersheds in China. To evaluate the vulnerability of terrestrial ecosystems to climate variability, the VSI was calculated based on the monthly mean–variance relationship and relative weights of vegetation and climate variables [57,58], as follows:

(1) $$\mathrm{VSI}=\sum^{\;}\left(TEM_{wei}\times TEM_{sen}+PRE_{wei}\times PRE_{sen}+RAD_{wei}\times RAD_{sen}\right)$$

where$\;TEM_{wei}$,$\;PRE_{wei}$, and $RAD_{wei}$ are the relative importance of climate variables to vegetation change (climate weights), respectively. $TEM_{sen}$, $PRE_{sen}$, and $RAD_{sen}$ are the sensitivity of vegetation to climate variables. Detailed methods are provided in Seddon et al. [56].

## 3. Results

### 3.1. Characteristics of Vegetation Variations

**Figure 2:**
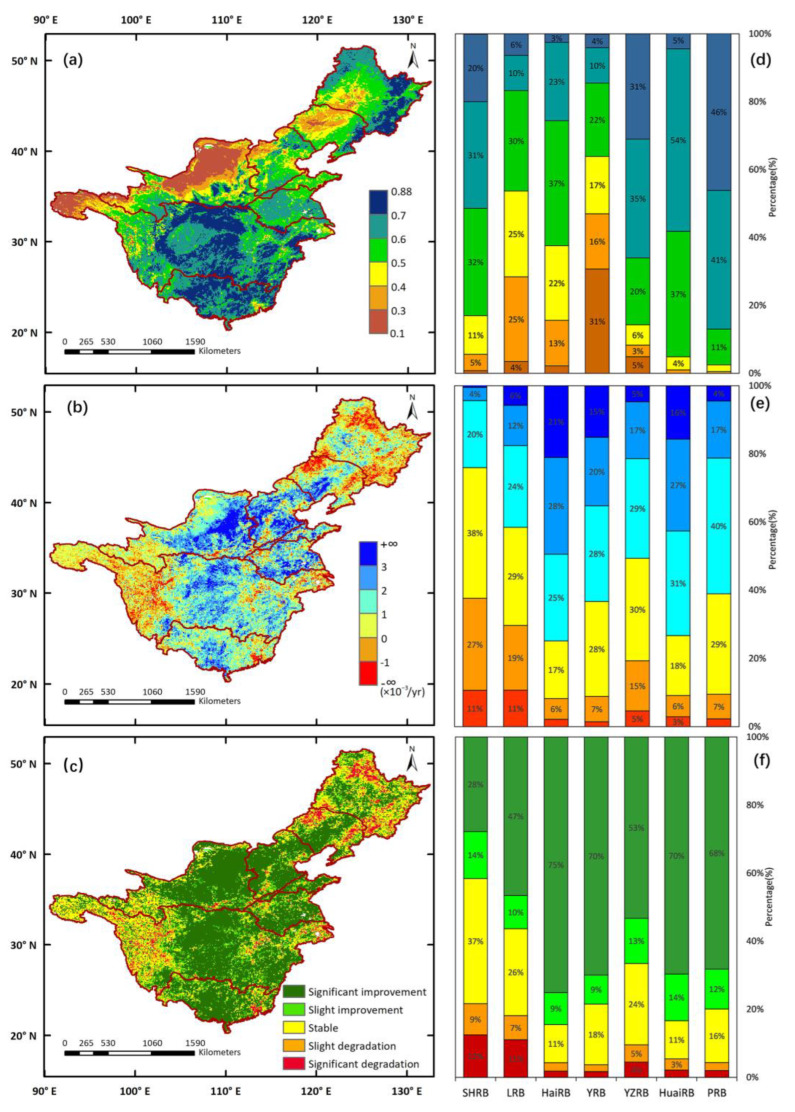
The spatial distribution of (**a**) mean annual growing season NDVI (GS-NDVI), (**b**) trends of GS-NDVI, (**c**) change degrees of GS-NDVI in seven major watersheds during 1982–2015, and the percentage distribution of (**d**) mean annual GS-NDVI, (**e**) trends, and (**f**) change degrees in each watershed.

To illustrate the general spatial variations of vegetation in the seven major watersheds, the average values, linear trends, and change degrees of GS-NDVI were analyzed (Figure 2a–c) from 1982 to 2015. The mean annual GS-NDVI in the seven major watersheds during 1982–2015 ranged from 0.01 to 0.88, increasing from the northwest to the south and southeast. As shown in Figure 2a, the mountainous and hilly regions, which are primarily composed of forests, grassland, and shrubland, in the central part of the YZRB and the entire PRB, showed relatively higher NDVI values than other areas. Meanwhile, lower NDVI values were revealed in the source regions of the YZRB and YRB (dominated by AMT), the Loess Plateau of the YRB (dominated by grassland), and the western parts of the SHRB and LRB (dominated by grassland and cropland). The changes in GS-NDVI revealed growing linear trends in most areas of the seven major watersheds, and the trends of some areas in HaiRB, HuaiRB, the northeast of the Loess Plateau, and the central parts of the YZRB and PRB had an increasing speed of over 3 × 10^−3^/yr. However, decreasing trends of vegetation were mostly identified in the source regions of the YZRB and YRB, the forest and grassland areas of the SHRB and LRB, the Yangtze River Delta, and the Pearl River Delta (Figure 2b). The spatial distribution of changing groups (Figure 2c) in the seven major watersheds during 1982–2015 confirmed the spatial patterns of the GS-NDVI trends displayed in Figure 2b. Pixels with significantly improved vegetation accounted for more than 50%, with 20% being stable, while only a few pixels showed degraded vegetation.

**Figure 3:**
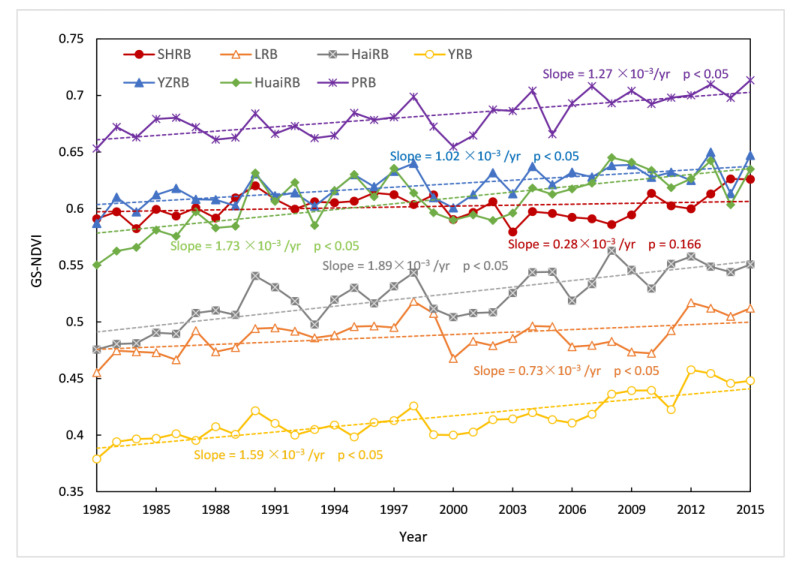
Inter-annual variation in the GS-NDVI in SHRB, LRB, HaiRB, YRB, YZRB, HuaiRB, and PRB from 1982 to 2015.

Moreover, to investigate the vegetation variations within each watershed, the inter-annual variation in the GS-NDVI (Figure 3), and the percentages of GS-NDVI averaged values, trends, and change degrees (Figure 2d−f) for each watershed were illustrated. The GS-NDVI in all watersheds presented positive linear trends, with most trends being significant (*p* < 0.05), except that of the SHRB. For the SHRB, the majority of GS-NDVI values in this basin ranged from 0.5 to 0.7, accounting for 63% of the total pixels. Around 85% of the pixels showed decreasing trends, of which 12% were significant. For the LRB, the linear trend of GS-NDVI was 0.73 × 10^−3^/yr, being smaller than that of other watersheds, except for the SHRB. In addition, around 80% of the pixels in the LRB had a mean annual GS-NDVI between 0.3 and 0.6, and 59% of them showed decreasing trends, with 12% being significant. For HaiRB, the growing trend of GS-NDVI showed a speed of 1.89 × 10^−3^/yr, being the largest trend among all watersheds. The mean annual GS-NDVI in HaiRB was evenly distributed between 0.3 and 0.7. The percentage of pixels with increasing trends was 93%, of which 75% were significant. As for the YRB, an increasing trend with the rate of 1.59 × 10^−3^/yr was identified, with 94% of pixels showing increasing trends and 70% being significantly improved, whereas most of the pixels had a mean annual GS-NDVI lower than 0.5, accounting for 64%. For the YZRB, we observed a rising trend of 1.02 × 10^−3^/yr in GS-NDVI, with 66% of pixels having a mean annual GS-NDVI greater than 0.6. The percentage of pixels with decreasing trends was 20%, while only 4% of them were significant. For HuaiRB, the trend of GS-NDVI was 1.73 × 10^−3^/yr. Almost 91% of pixels had a mean annual GS-NDVI between 0.5 and 0.6, and also 91% of pixels showed increasing trends, with 70% being significant. For the PRB, we observed a rising trend at the rate of 1.27 × 10^−3^/yr, with 87% pixels having mean annual GS-NDVI values greater than 0.6, and the percentage of increasing trends and significantly improved change types was 91% and 68%, respectively.

### 3.2. Regionalization of GS-NDVI Variations

The hybrid regionalization approach was performed on the monthly GS-NDVI to further explore the spatial anomalies and clustering of GS-NDVI over the seven major watersheds. Table 2 summarizes the variance contribution of the retained EOFs before rotation, and the first nine EOFs of GS-NDVI were retained, with a cumulative explained variance of over 85.1%. Meanwhile, according to North’s Rule of Thumb, all EOFs were statistically significant. The positions with the largest absolute values of RLV after the Varimax rotation were chosen as the initial clustering centers.

**Figure 4:**
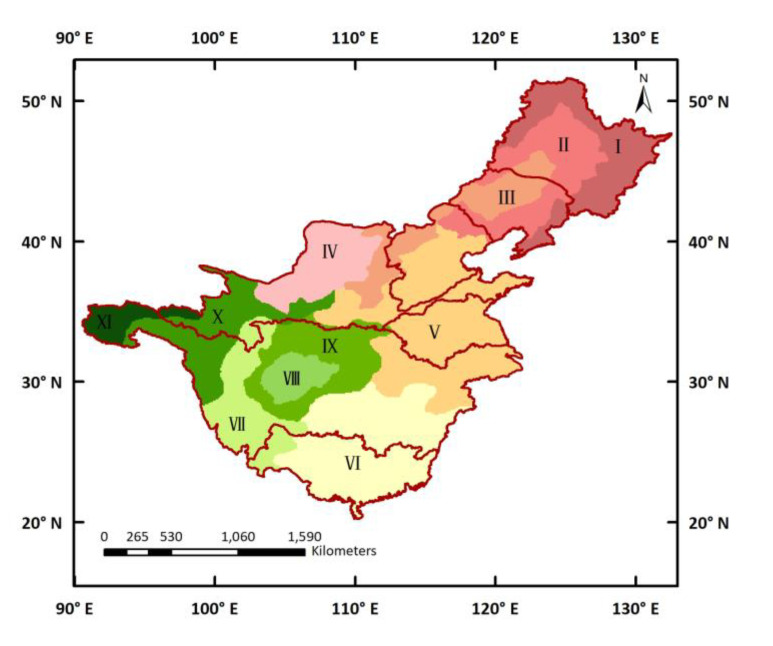
Regionalization of GS-NDVI obtained by a hybrid regionalization approach over seven major watersheds during 1982–2015.

With the K-means clustering algorithm, all pixels in the seven major watersheds were clustered into nine groups using pre-defined cluster numbers and centroids. However, as the spatial feature was not taken into account during cluster analysis, the pixels in each cluster were non-contiguous. Finally, based on the cluster groups and change patterns of GS-NDVI, the homogenous regions were identified as 11 groups (Figure 4). At the same time, the percentages of the main vegetation types in each sub-region are listed in Table 3. It should be particularly pointed out that the division of sub-regions was mainly based on the topography and vegetation types. Region Ⅰ was predominately located in the Greater Khingan Mountains, Lesser Khingan Mountains, and Changbai Mountains, and mainly consisted of BF, cropland, and NF. This region was characterized by higher values and stronger variations in GS-NDVI. Regions Ⅱ, Ⅲ, and Ⅳ covered the Northeast China Plain, Taihang Mountains, and Loess plateau. The main vegetation types in these three sub-regions were cropland and grassland, with the proportion of cropland gradually decreasing, and the proportion of grassland gradually increasing. The North China Plain and the plains in the middle and lower reaches of the YZRB were categorized as Region Ⅴ, with 70% being cropland. The southeastern hilly area in China was the main part of Region Ⅵ, which mainly consisted of NF, shrubland, and cropland. Moreover, Region Ⅷ represented the cropland-based Sichuan Basin, whereas mountainous areas with forests and shrubland were considered as Region Ⅸ. In addition, the source regions of the YZRB and YRB were divided into three sub-regions (Regions Ⅶ, Ⅹ, Ⅺ), consisting of the central Tibetan Plateau, the Hengduan Mountains, and the Yunnan-Guizhou Plateau. The main vegetation types of these three sub-regions were NF and shrubland, shrubland and AMT, and grassland and AMT, respectively.

**Figure 5:**
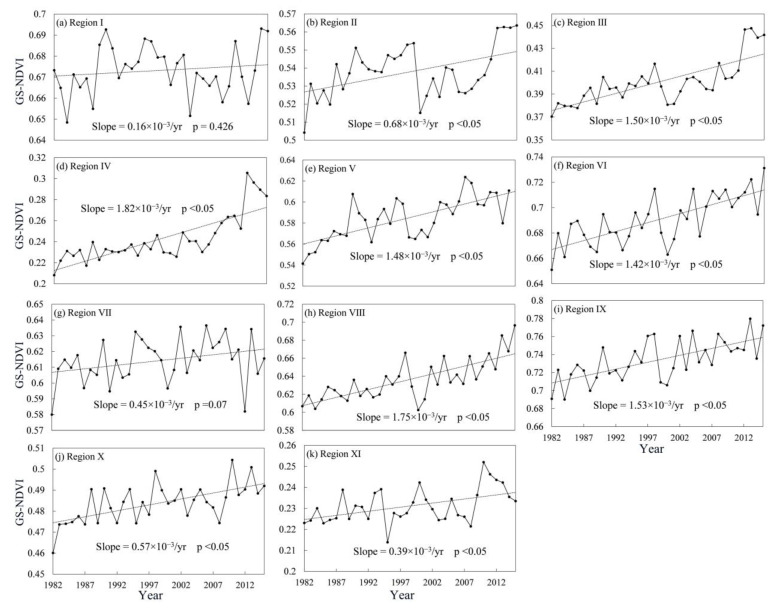
Inter-annual variation in the GS-NDVI in 11 sub-regions during 1982–2015.

**Figure 6:**
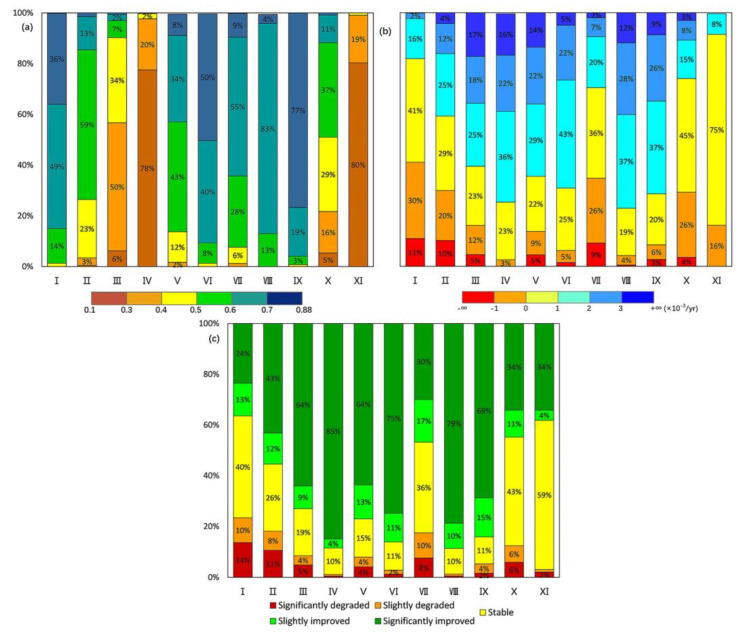
The percentage distribution of (**a**) mean annual GS-NDVI, (**b**) trends, and (**c**) change degrees in 11 sub-regions during 1982−2015.

Based on the results of the 11 homogenous sub-regions, the inter-annual variations, average values, linear trends, and change degrees of GS-NDVI were analyzed from 1982 to 2015 for each sub-region (Figure 5 and Figure 6). Compared with the heterogeneity of vegetation dynamics at the watershed scale, the characteristics of vegetation change among the 11 sub-regions were more homogeneous. The regionally averaged GS-NDVI for 1982–2015 showed significant positive trends for most of the sub-regions at a confidence level of 95%, except Region Ⅰ and Ⅶ. Among these regions, the increasing trends in Region Ⅳ and Ⅷ showed great speeds, with slopes of approximately 1.82 × 10^−3^/yr and 1.75 × 10^−3^/yr, respectively. As shown in Figure 6, the mean annual GS-NDVI values in most of the pixels in Regions Ⅰ, Ⅵ, Ⅷ, and Ⅸ were greater than 0.6, while most values for Regions Ⅲ, Ⅳ, and Ⅺ fell between 0.1 and 0.4. Overall, significantly improved vegetation was the main change type in most sub-regions, while, for Regions Ⅰ, Ⅶ, Ⅹ, and Ⅺ, the majority of changes were of the stable type. Furthermore, there was still a certain percentage of pixels with significantly degraded vegetation in Regions Ⅰ, Ⅱ, Ⅶ, and Ⅹ, which accounted for 14%, 11%, 8%, and 6%, respectively.

### 3.3. Vegetation Sensitivity to Climate Variables

**Figure 7:**
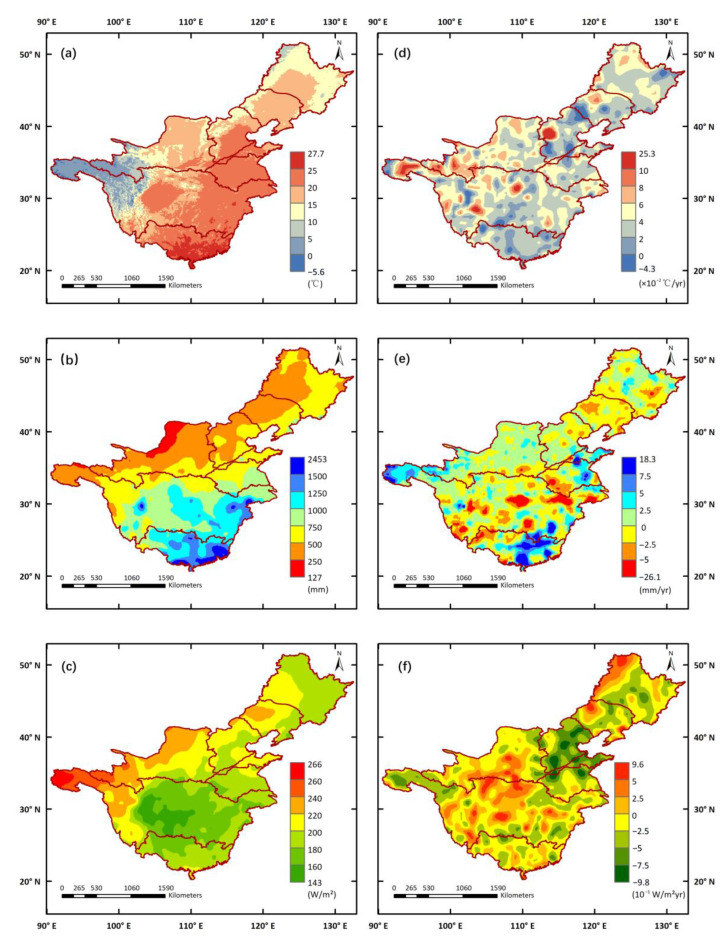
The spatial distribution of climate variables in seven major watersheds during 1982–2015: (**a**) mean annual GS-TEM, (**b**) mean annual GS-PRE, (**c**) mean annual GS-RAD, and their change trends (**d**–**f**).

Figure 7 illustrates the spatiotemporal variations in climate variables in the growing season during 1982–2015. The mean annual GS-TEM across the seven major watersheds was between −5.6 and 27.7 °C, and the distribution of GS-TEM was substantially consistent with the altitude. The GS-PRE showed an increasing gradient from the northwest to the south and southeast, which was similar to that of the GS-NDVI. The higher values of mean annual GS-TEM and GS-PRE both occurred in the coastal areas of the PRB (Figure 7a,b). However, the GS-RAD had the opposite spatial distribution to GS-TEM and GS-PRE, with the maximum and minimum values occurring at the source regions of the YZRB and YRB and the Sichuan Basin in the central part of the YZRB, respectively (Figure 7c). The changing trends of these variables (Figure 7d–f), however, did not show obvious spatial characteristics similar to those of the mean values during 1982–2015. The increasing GS-TEM was detected in most pixels of the seven major watersheds, and the relatively high variability of GS-TEM appeared in the northeast of HaiRB, the source regions of the YZRB and YRB, and the central part of the YZRB. For GS-PRE, both increasing and decreasing trends were revealed in the seven major watersheds, and more than 70% of the pixels ranged from −2.5 to 2.5 mm/yr. The most dramatic decreasing trends of GS-PRE mainly appeared in the central and lower parts of the YZRB, while obvious increasing tendencies were found in the source regions of the YZRB, parts of the YRB and HuaiRB, and parts of the YZRB and PRB. The change trends in GS-RAD ranged from −0.98 to 0.96 W/m^2^yr, with decreasing trends most often located in the northeastern areas of the seven major watersheds, except the Greater Khingan Mountains. Furthermore, there was no consistency between the spatial distribution of GS-NDVI trends and the trends of climate variables (Figure 7d–f), indicating that vegetation change was not controlled by an individual climate variable, but was more likely influenced by a comprehensive effect of these variables.

**Figure 8:**
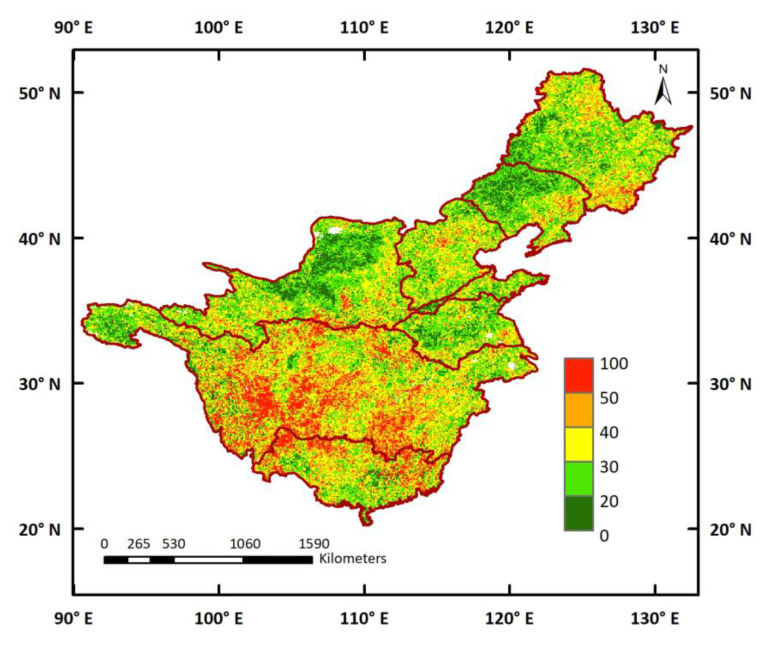
Spatial distributions of Vegetation Sensitivity Index (VSI) in seven major watersheds during 1982–2015.

**Figure 9:**
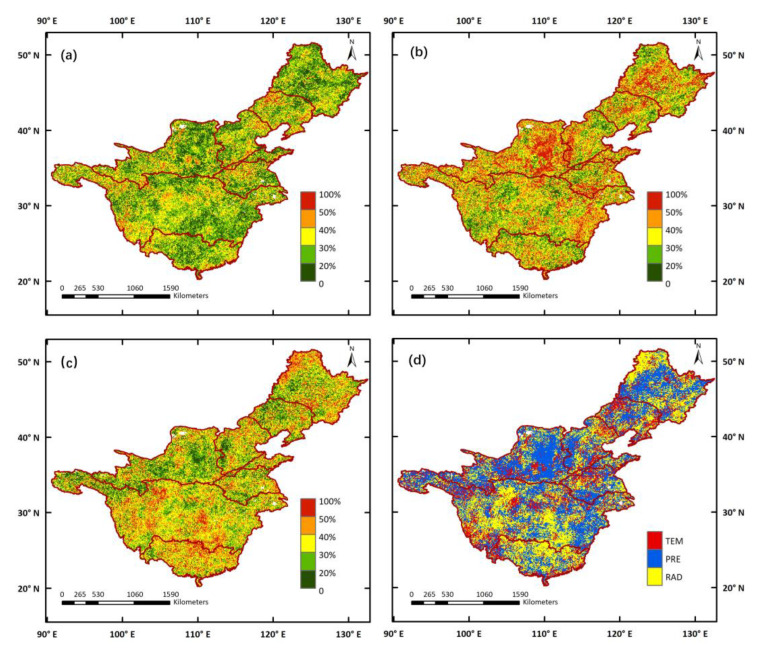
Spatial distributions of contributions of climate variables to VSI: (**a**) TEM, (**b**) PRE, (**c**) RAD, and (**d**) the primary controlling climate variable of VSI.

To identify the comprehensive effect of climate variables, the VSI was used to quantify the sensitivity of terrestrial ecosystems to climate. The VSI distribution revealed prominent spatial discrepancies (Figure 8). The central parts of the YZRB showed high VSI values, with vegetation types dominated by forests, shrubland, and cropland, which indicated high sensitivity to climate variables in this area. Correspondingly, lower VSI values emerged predominantly in the source regions of the YZRB and YRB, the Loess Plateau of the YRB, and the western part of the LRB, corresponding to the distribution of alpine meadows and tundra (AMT) and grassland. The relative contributions of each climate variable to the VSI are displayed in Figure 9. Overall, in most of the study areas, temperature contributed less than 40% to the VSI and there were only a few areas where the VSI appeared primarily controlled by the temperature. Precipitation contributed more than 30% in most areas, and, in some pixels, the contributions were higher than 50%. In total, more than 45% of the pixels of the VSI were controlled by precipitation, mostly located in the central parts of the SHRB and LRB, the Loess Plateau of the YRB, and the lower part of the YZRB. The contributions of solar radiation to the VSI were relatively higher at low latitudes than at high latitudes, as it was revealed as the dominant factor in the central parts of the YZRB and PRB. As an exception, in the northwest of the SHRB, solar radiation was still a dominant factor in the VSI, despite the high altitude.

Moreover, to quantify the sensitivity of climate variables on GS-NDVI in homogenous regions, the trends of climate variables, the VSI, and the contributions of each climate variable for each sub-region were determined, and results are given in Table 4. Significant positive trends in GS-TEM were found in all sub-regions, while the trends of GS-PRE were significantly negative. The smallest changing slopes for GS-TEM and GS-PRE were revealed in Region Ⅵ, with a speed of 0.02 °C/yr and −6.97 mm/yr, respectively. As for GS-RAD, significant negative trends were identified in Regions Ⅱ, Ⅲ, Ⅴ, and Ⅺ, while in other sub-regions, only weak decreasing trends or even slightly increasing trends were found. The higher VSIs occurred in Regions Ⅵ, Ⅶ, Ⅷ, and Ⅸ, covered mainly by BF, NF, shrubland, and cropland, while Regions Ⅱ, Ⅲ, Ⅴ, and Ⅺ, covered by grassland, ATM, and cropland, had relatively lower VSIs. The relative contributions of three climate variables (TEM, PRE, RAD) to vegetation sensitivity varied among sub-regions: vegetation in Regions Ⅱ, Ⅲ, Ⅳ, Ⅴ, Ⅹ, and Ⅺ was more sensitive to precipitation variability, whereas vegetation in Regions Ⅰ, Ⅵ, Ⅶ, Ⅷ, and Ⅸ was primarily influenced by solar radiation. Notably, although temperature was not the most important factor for vegetation sensitivity in any sub-region, it was still non-negligible as it accounted for certain contributions.

## 4. Discussion

This is the first comprehensive study to explore vegetation–climate relationship patterns across watersheds in China. Our results are valuable for understanding the evolutionary mechanisms of hydrological processes, which provide the foundation for water resource planning and management [3,36]. This study provides detailed information on vegetation changes in China, with several outcomes.

First, differing from previous studies that mostly focused on individual basins [38,40], this study analyzed the characteristics of seven major watersheds to understand the overall situation of watersheds in China. It can be seen that the increasing trends of GS-NDVI in HaiRB and HuaiRB were more rapid than in other watersheds (Figure 1). By contrast, the linear trends of GS-NDVI in the SHRB and LRB were positive but slight, with the largest proportion of vegetation being significantly degraded (Figure 2). However, there are still some studies that are inconsistent with the results presented in this paper. For example, it is clear from Figure 2f that 70% of the vegetation in the YRB was significantly improved and vegetation in a few pixels was degraded, whereas Jiang et al. [53] found that 32.8% of areas in the YRB with vegetation significantly improved, while 27.7% were significantly degraded. The discrepancy is mainly because the NDVI used in these two studies was derived from the GIMMS NDVI3g dataset and MOD13A2 NDVI product data, and the spatial and temporal revolutions of the two datasets were not identical. Tian et al. [59] assessed vegetation greening trends, generated from different NDVI datasets, in the YRB and concluded that the GIMMS dataset is commonly applied to explore large-scale vegetation change because its time series is long and continuous [14,60]; correspondingly, MODIS data are frequently employed in monitoring dynamic vegetation at regional scales [61].

Another outcome of this study was the use of the hybrid regionalization approach to identify the homogenous regions in seven major watersheds across China. Compared with administration and geography boundaries [62,63], homogeneous sub-regions obtained by clustering algorithms can capture more spatial information. An example is the fact that the Loess Plateau was identified as an independent sub-region (Region IV) with a low mean annual GS-NDVI and high variability. This is in accordance with previous studies suggesting that the Loess Plateau is a unique area, where several afforestation programs have been launched by the Chinese government over the past few decades [64]. Furthermore, the results of regionalization demonstrated that the vegetation type and topography are important factors associated with the spatial patterns of vegetation variations [65]. On one hand, different vegetation types have different vegetation–climate relationships, resulting in different changing patterns in vegetation [66], which makes it reasonable to evaluate vegetation changes and their link with climate within different ecosystems [47]. On the other hand, topographic attributes, including elevation, slope, and aspect, introduce heterogeneity into the effects of water availability, radiation, and temperature on vegetation greenness [44,67,68]. It can be seen in Figure 4 that the source regions of the YZRB and YRB were divided into three sub-regions (Regions VII, X, XI) by elevation. This finding has also been confirmed by previous studies [45,69] stating that vegetation responses to climate conditions in the Qinghai–Tibet Plateau are elevation-dependent.

Third, this study provided an interpretation of vegetation sensitivity by vegetation type. Our results showed that the VSIs in the central parts of the YZRB, covered by forests, shrubland, and cropland, were higher than those in the source regions of the YZRB and YRB, the Loess Plateau of the YRB, and the western parts of the SHRB and LRB, covered by AMT and grassland (Figure 8). This suggests that vegetation types such as forests and shrubland are relatively more vulnerable to climate variations than grassland and AMT. Other studies have also documented that vegetation dominated by trees and tall shrubs is more responsive to climate change than dwarf shrub vegetation [70,71], and temperature-induced drought stress has a greater inhibitory effect on tree activity than shrubs [71]. As illustrated in Figure 9, vegetation in arid and semi-arid regions showed strong responses to precipitation, including the central part of the SHRB and LRB and the Loess Plateau of the YRB; meanwhile, vegetation activities in the central part of the YZRB and PRB were more sensitive to solar radiation variations. These findings are generally consistent with those of Ge et al. [27], Papagiannopoulou et al. [72], and Seddon et al. [56], taking into consideration the diverse research periods and the differences in methodology and data. Meanwhile, the weaker association between vegetation and temperature has also been confirmed by Piao et al. [73], where the intensity of the link between vegetation and temperature decreased significantly in the Northern Hemisphere. Nevertheless, there are still other regional-scale studies indicating that temperature has a greater impact on vegetation than precipitation [74], likely because these studies only focused on the response of vegetation to temperature and precipitation in the mean climate state, ignoring how complex vegetation responds to the changing climate and the importance of solar radiation.

Overall, this study combined a hybrid regionalization approach and the VSI to better understand the changing patterns of vegetation and its sensitivity to climate variability across watersheds in China, providing new perspectives on vegetation changes and the triggering mechanism of climate change. However, some limitations and uncertainties cannot be neglected. First, the time lag and cumulative effects of climate on vegetation should not be ignored [3,75,76]. Second, the vulnerability of vegetation to climate variability, as evaluated by the VSI, was investigated using linear models, while neglecting the fact that actual vegetation–climate connections may be complex and nonlinear [72,77]. Lastly, other driving factors, such as CO_2_ and human activities, which are also considered essential factors in vegetation growth [63,78] were not included in this study. Thus, future studies will address the questions above and explore further vegetation variations.

## 5. Conclusions

Understanding vegetation dynamics at the watershed scale can benefit the planning, management, and sustainable development of water resources. In this study, the changing patterns of vegetation and its sensitivity to climate variability across watersheds in China were investigated based on a hybrid regionalization approach and the VSI. The GS-NDVI was significantly improved in more than 50% of the seven major watersheds, while only a few areas showed degradation. The GS-NDVI in all watersheds presented a positive linear trend, with most trends being significant, except that of the SHRB. The entire study area was categorized into 11 sub-regions, and the regionalization results were in good agreement with the distributions of topography and vegetation types. Compared with the heterogeneity of vegetation dynamics at the watershed scale, the characteristics of vegetation change among the 11 sub-regions were more homogeneous. With the exception of Regions I, VII, X, and XI, significantly improved vegetation was the main change type in most sub-regions. The central parts of the YZRB showed higher VSIs, while lower VSIs emerged predominantly in the source regions of the YZRB and YRB, the Loess Plateau of the YRB, and the western part of the LRB. The VSI was highest in Regions VII, VIII, and IX, and lowest in Region IV. Moreover, the relative contributions of three climate variables to vegetation sensitivity varied among sub-regions.

## Figures and Tables

**Table 1:** Detailed information on seven major watersheds in China.

	SHRB	LRB	HaiRB	YRB	YZRB	HuaiRB	PRB
Longitude range (°E)	119.9–132.5	116.5–125.8	112–120	95.8–119.1	90.6–122.4	111.9–121.4	102.2–115.9
Latitude range (°N)	41.7–51.6	38.7–45	35–43	32.1–41.8	24.5–35.8	30.8–36.6	21.5–26.8
Watershed area (km^2^)	557,200	219,000	318,200	752,400	1,800,000	269,000	453,600
Climate characteristic	Temperate humid and semi-humid monsoon climate	Temperate semi-humid and semi-arid monsoon climate	Temperate semi-humid and semi-arid monsoon climate	Temperate humid, semi-humid, and semi-arid continental monsoon climate	Subtropical humid, semi-humid, and semi-arid monsoon climate	Subtropical and temperate semi-humid monsoon climate	Subtropical humid monsoon climate

**Table 2:** Variance contribution (%) of the retained EOFs before rotation, and the difference between the spacing of adjacent eigenvalues (Δs) and the sampling error (Δλ), for the retained EOFs tested by North’s Rule of Thumb.

	EOF1	EOF2	EOF3	EOF4	EOF5	EOF6	EOF7	EOF8	EOF9
% of variance	58.2	9.3	5.7	4.4	2.8	1.7	1.3	1.0	0.7
Cumulative %	58.2	67.5	73.1	77.5	80.3	82.0	83.3	84.4	85.1
Δs − Δλ	3600.8	262.5	89.2	116.1	77.3	23.7	22.8	21.1	9.9

**Table 3:** Percentages of main vegetation types (broadleaf forest (BF), mixed forest (MF), needleleaf forest (NF), shrubland, grassland, alpine meadows and tundra (AMT), cropland, swamp) in each sub-region.

Vegetation	Ⅰ	Ⅱ	Ⅲ	Ⅳ	Ⅴ	Ⅵ	Ⅶ	Ⅷ	Ⅸ	Ⅹ	Ⅺ
BF	45%	10%	10%		5%	7%	5%		22%	3%	
MF	3%										
NF	12%		2%		6%	25%	26%	9%	12%	6%	
Shrubland	3%	9%	8%	3%	5%	27%	26%	2%	27%	21%	
Grassland	8%	20%	35%	58%	6%	12%	13%	4%	8%	11%	31%
AMT							13%			49%	68%
Cropland	21%	57%	46%	37%	77%	28%	16%	85%	29%	11%	
Swamp	6%	3%									

**Table 4:** Trends of climate variables, Vegetation Sensitivity Index, and climate variable contributions in each sub-region during 1982–2015.

	Trends			Vegetation Sensitivity Index			
	TEM(°C/yr)	PRE(mm/yr)	RAD(W/m^2^·yr)	VSI	TEM (%)	PRE (%)	RAD (%)
Region I	0.035 *	−3.60 *	−0.012	32.7	29%	34%	38%
Region II	0.032 *	−3.07 *	−0.230 *	28.7	29%	38%	33%
Region III	0.043 *	−2.48 *	−0.242 *	26.9	28%	42%	31%
Region IV	0.046 *	−1.84 *	−0.061	23.6	26%	43%	31%
Region V	0.037 *	−4.55 *	−0.314 *	31.3	29%	39%	33%
Region VI	0.027 *	−6.97 *	−0.126	38.5	27%	35%	38%
Region VII	0.039 *	−4.89 *	−0.048	42.7	32%	33%	35%
Region VIII	0.044 *	−5.93 *	0.096	40.6	32%	33%	35%
Region IX	0.042 *	−5.46 *	0.179	41.6	29%	35%	37%
Region X	0.053 *	−3.02 *	−0.116	31.8	30%	38%	32%
Region XI	0.052 *	−1.73 *	−0.339 *	26.9	31%	40%	29%

Note: * indicates significant trends at the confidence level of 95%.

## Data Availability

Publicly available datasets were analyzed in this study. The NDVI dataset can be found at http://ecocast.arc.nasa.gov/data/pub/gimms (accessed on 12 February 2020); the CMFD dataset can be found at https://data.tpdc.ac.cn/en/data/8028b944-daaa-4511-8769-965612652c49 (accessed on 20 February 2020); the vegetation type data can be found at https://www.resdc.cn (accessed on 8 March 2020).

## Abbreviations

Vegetation Sensitivity Index (VSI); Songhua River Basin (SHRB); Liao River Basin (LRB); Hai River Basin (HaiRB); Yellow River Basin (YRB); Yangtze River Basin (YZRB); Huai River Basin (HuaiRB); Pearl River Basin (PRB); normalized difference vegetation index (NDVI); growing season NDVI (GS-NDVI); China Meteorological Forcing Dataset (CMFD); broadleaf forest (BF); broadleaf and needleleaf mixed forest (MF); needleleaf forest (NF); alpine meadows and tundra (AMT); rotated loading vector (RLVs); Rotated Empirical Orthogonal Function (REOF); temperature (TEM); precipitation (PRE); solar radiation (RAD).
